# Data on synthesis and characterization of sulfonated poly(phenylnorbornene) and polymer electrolyte membranes based on it

**DOI:** 10.1016/j.dib.2019.104626

**Published:** 2019-10-07

**Authors:** Oleg S. Morozov, Boris A. Bulgakov, Anna V. Ivanchenko, Svetlana S. Shachneva, Sergey S. Nechausov, Maxim V. Bermeshev, Alexey V. Kepman

**Affiliations:** a Lomonosov Moscow State University, Department of Chemistry, 119991, Leninskie Gory, 1-3, Moscow, Russia; b Lomonosov Moscow State University, Faculty of Materials Science, 119991, Leninskie Gory, 1-73, Moscow, Russia; c A.V. Topchiev Institute of Petrochemical Synthesis, Russian Academy of Sciences, 117912, Leninskiy prosp. 29, Moscow, Russia

**Keywords:** Sulfonated polymer, Ionomer, Ionic liquids, Polyelectrolyte membrane, Ionic conductivity

## Abstract

This article describes data on preparation of sulfonated hydrogenated poly(phenylnorbornene) with different cations synthesized via sequential ring-opening metathesis polymerization, reduction, homogeneous sulfonation and cation exchange reactions. The data of the characterization of new polymers by nuclear magnetic resonance (^1^H NMR) spectroscopy, Fourier-transform infrared (FT-IR) spectroscopy and differential scanning calorimetry (DSC) are presented. The effect of imidazolium and 1-methylimidazolium cations, ionic liquid and Zwitter-type ion liquid on ionic conductivities evaluated by impedance spectroscopy. Preparation procedure of polymer electrolyte membrane based on new polymers and Nafion as a blend with polyvinylidene fluoride (PVDF) is given. Scanning electron microscopy images and ionic conductivities of these membrane are presented.

**Table:** Specifications Table

Subject	Materials Sciences, Chemistry
Specific subject area	Polymer electrolyte membrane
Type of data	Table, Image, Figure, Text describing synthesis
How data were acquired	Nuclear magnetic resonance (NMR) and Fourier-transform infrared (FT-IR) data were proceeded using ACD/SpecManager software Ver. 10.00. DSC was proceeded using Universal Analysis 2000 software Ver. 4.5A. Ionic conductivity values were proceeded using ES8 software ver. 4.189 ^1^H NMR spectra were acquired at 600 MHz on a Bruker Avance III Ultrashield spectrometer. The FTIR spectra were acquired in the range of 4000–400 cm^−1^ on Bruker Tensor-27 spectrophotometer using KBr pellets. The differential scanning calorimetry (DSC) data were obtained on a TA Instruments Q20 in a sealed aluminum pan with a heating rate of 10 °C/min under N_2_ purge. Scanning electron microscopy (SEM) was performed on an a TESCAN Vega 3 instrument with an accelerating voltage of 20 kV. The samples were sputter-coated with approximately 10 nm of gold before analysis. Electrochemical impedance spectroscopy (EIS) was conducted on Electro Chemical Instruments P-45× potentiostat with impedance module FRA-24 M.
Data format	Raw Analyzed
Parameters for data collection	All synthetic procedures were carried out using standard Schlenk techniques under an argon atmosphere. All solvents were analytical grade and were purchased from local suppliers (EKOS-1, Chimmed, Russia). Other chemicals were purchased from commercial sources (Acros, Aldrich). All measurements were carried out at room temperature according to standard methods.
Description of data collection	Ionic conductivities of membranes with variable ion conducting media, variable polymer and variable cations of sulfo groups were calculated from Nyquist plots. For each membrane the impedance data was obtained in 5–7 different parts of the films. Ionic conductivity were calculated for each experiment, average value and standard deviation are provided.
Data source location	Moscow, Russian Federation
Data accessibility	Analysed data are available with the article. All raw data have been deposited in the public repository. Repository name: Mendeley Data Data identification number: https://doi.org/10.17632/wcx58k2rp2.1 Direct URL to data: https://data.mendeley.com/datasets/wcx58k2rp2/1

**Table:** 

**Value of the data** • The impedance spectroscopy data indicate that the exchange of cations has a significant effect on the ionic conductivity of sulfonated polymers • The data obtained will be useful for the development of non-aqueous ionic membranes for fuel cells, gas separation, polymer sensors and actuators • Described synthetic procedure and obtained spectral data will be useful for preparation and structure elucidation of new sulfonated hydrocarbon polymers

## 1 Data

**Scheme 1:**
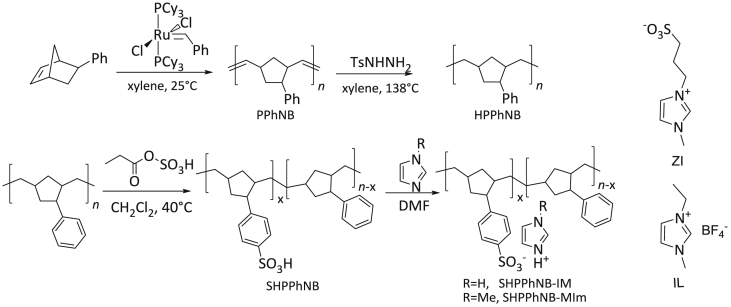
Synthesis of sulfonated hydrogenated poly(phenyl norbornene) and cation exchange reactions.

**Fig. 1:**
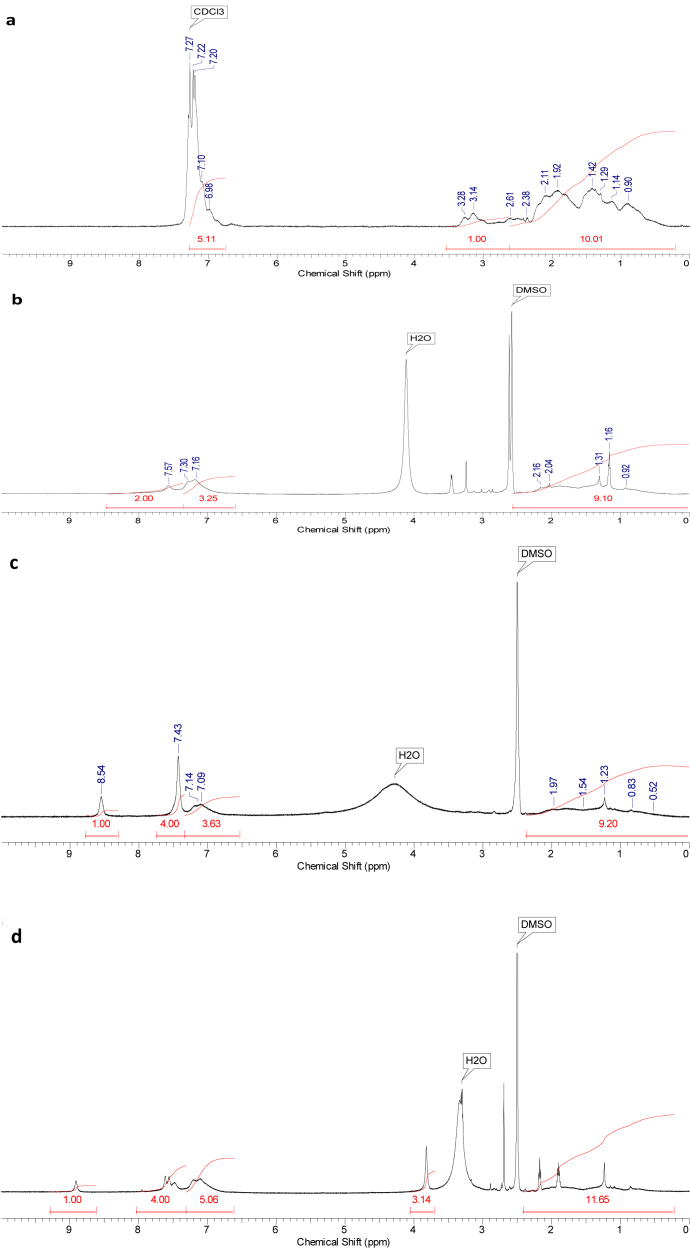
^1^H NMR spectra of HPPhNB (a), SHPPhNB (b), SHPPhNB-Im (c) and SHPPhNB-MIm (d).

**Fig. 2:**
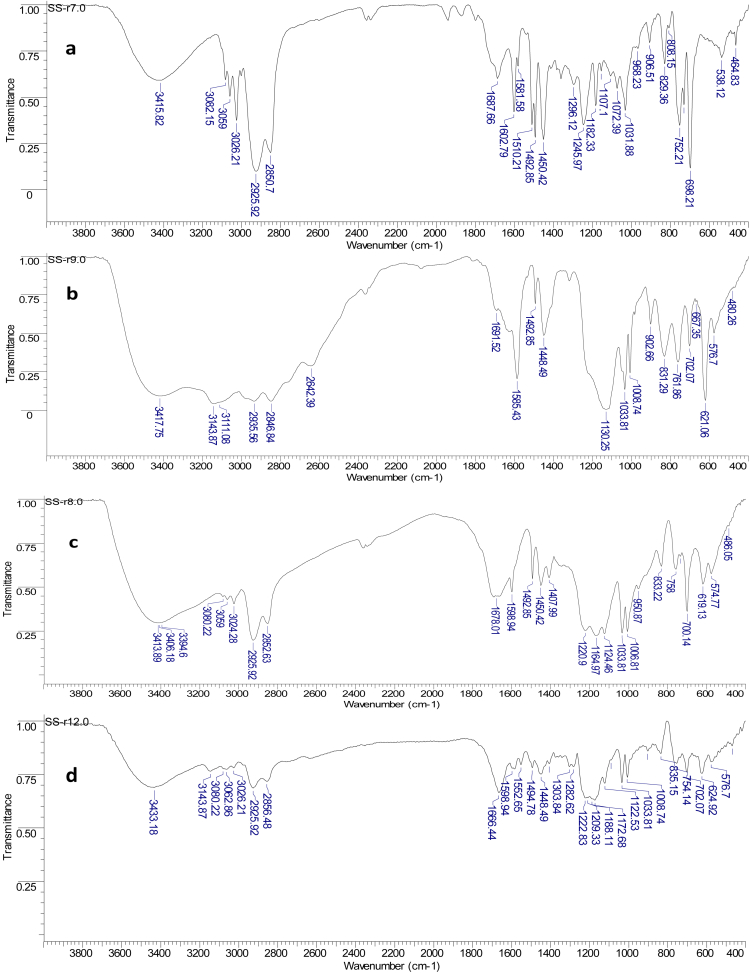
FT-IR spectra of HPPhNB (a), SHPPhNB (b), SHPPhNB-Im (c) and SHPPhNB-MIm (d).

**Fig. 3:**
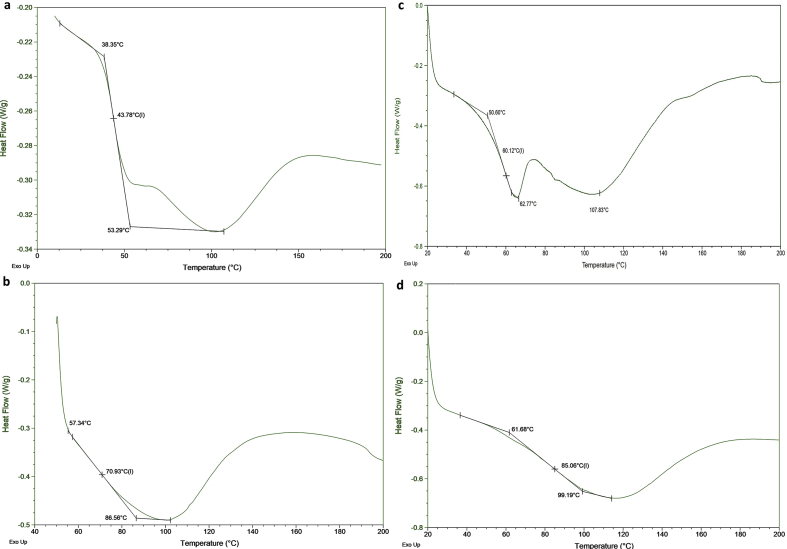
DSC curves of HPPhNB (a), SHPPhNB (b), SHPPhNB-Im (c) and SHPPhNB-MIm (d).

**Fig. 4:**
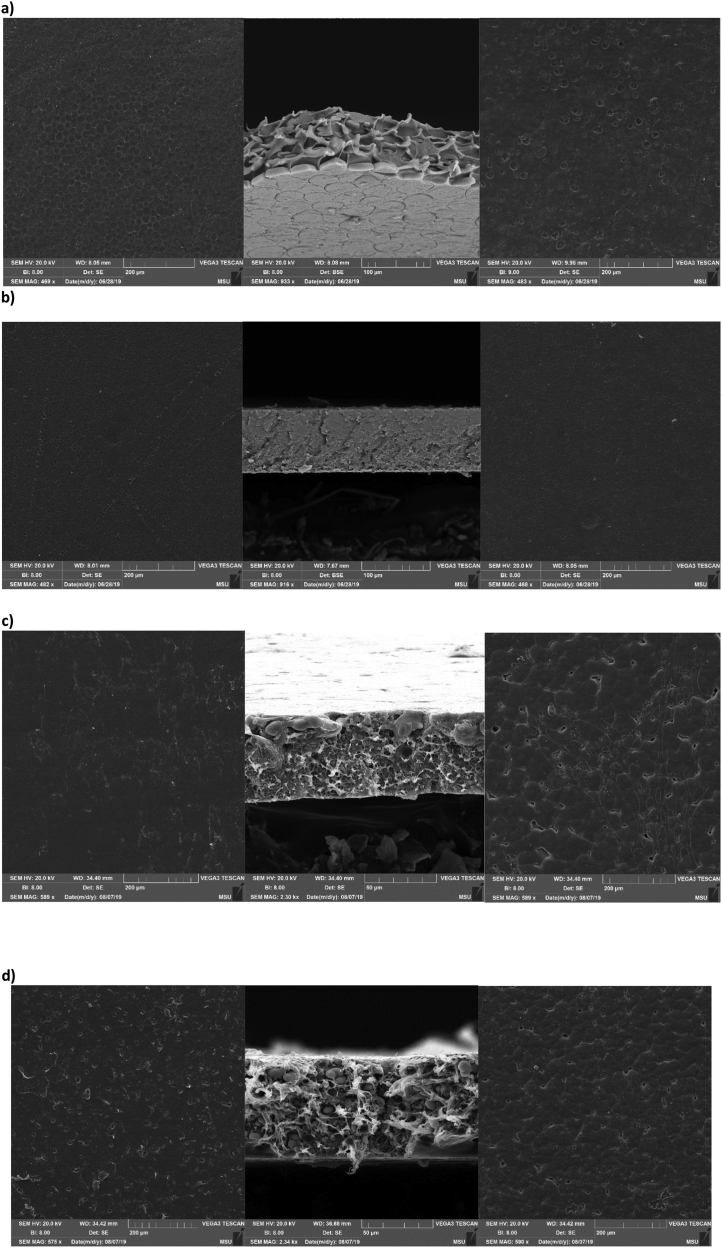
SEM images of bottom surface (left), cross-section (middle) and top surface (right) of PVDF/SPPhNB-Im/IL (a), PVDF/SPPhNB-MIm/IL (b), PVDF/Nafion-Im/IL (c), PVDF/Nafion-MIm/IL (d).

Perfluorosulfonic acid membranes such as Nafion are the most commonly used for polymer electrolyte membrane manufacturing. Many studies have focused on the search for new polymers that can replace Nafion membranes [1,2]. Sulfonated commercial available polymers such as sulfonate polystyrene [3], sulfonated poly(ether ether ketone) [4], sulfonated poly(styrene-ran-ethylene) [5], sulfonated poly(styrene-b-ethylene-co-butylene-b-styrene) [[6], [7], [8]], sulfonated polyetherimide [9], sulfonated polyimide [10] sulfonated styrenic pentablock copolymer [[11], [12], [13]], sulfonated polyphenylsulfone [14], sulfonated poly(1,4-phenylene ether-ether-sulfone) [15] were employed to prepare ionic actuators. Recently, highly conductive sulfonated polymer based polynorbornene prepared using ring-opening metathesis polymerization (ROMP) was described for preparation of proton exchange membrane [16]. This article describes the synthesis of hydrated poly(5-phenyl norbornene), its sulfonation and cation exchange procedures (Section 2.1,Scheme 1). The ^1^H NMR spectra (Fig. 1), FT-IR spectra (Fig. 2) and DSC curves (Fig. 3) of all polymers are shown in Section 2.2. Data on preparation of the polymer electrolyte membranes with using different ion conducting media (Section 2.3.1) and PVDF blend with synthesized polymers and Nafion™ (Section 2.3.2) are described. Data on ion conductivity (Table 1 and Table 2) are presented in Section 2.3. SEM images of both surfaces and cross-section of the blend membrane are shown in Fig. 4.

**Table 1:** Ion conductivity of sulfonated polymer with different cations and ion conducting media.

Membrane	Composition, %	Thickness, μm	Ionic conductivity, mS/cm
SPPhNB	100	83	0.014 ± 0.0086
SPPhNB-Im	100	196	0.041 ± 0.0078
SPPhNB-MIm	100	190	0.10 ± 0.028
SPPhNB/IL	50/50	173	1.39 ± 0.020
SPPhNB-Im/IL	50/50	180	3.44 ± 0.074
SPPhNB-MIm/IL	50/50	183	4.78 ± 0.16
SPPhNB-Im/IL/ZI	50/25/25	158	0.25 ± 0.091
SPPhNB-MIm/IL/ZI	50/25/25	160	0.44 ± 0.051

**Table 2:** Mechanical properties and ion conductivity of blend membranes.

Membrane	Composition, %	Thickness, μm	Ionic conductivity, mS/cm
PVDF/SPPhNB-Im/IL	50/25/25	115	0.41 ± 0.062
PVDF/SPPhNB-MIm/IL	50/25/25	126	1.24 ± 0.087
PVDF/Nafion-Im/IL	50/25/25	102	0.71 ± 0.083
PVDF/Nafion-MIm/IL	50/25/25	125	0.69 ± 0.05

The raw data on characterization of the polymers and membranes are provided In Ref. [17]. The following data is presented:

a. ^1^H NMR free induction decay files of HPPhNB, SHPPhNB, SHPPhNB-Im and SHPPhNB-MIm
b. FTIR raw spectra of HPPhNB, SHPPhNB, SHPPhNB-Im and SHPPhNB-MIm
c. DSC curves of HPPhNB, SHPPhNB, SHPPhNB-Im and SHPPhNB-MIm
d. The data obtained by impedance electrochemical spectroscopy (EIS), which were used to calculate the ionic conductivities presented in Table 1, Table 2
e. Full-size SEM images of membranes

## 2 Experimental, materials and methods

### 2.1 Polymer synthesis

#### 2.1.1 Materials

5-Phenyl-2-norbornene was synthesized by the Diels-Alder reaction from styrene and dicyclopentadiene using the standard procedure [18]. 1-Ethyl-3-methylimidazolium tetrafluoroborate (IL) was synthesized by literature procedure [19]. Zwitter-type ion liquid 3-(1-methyl-3-imidazolio)propanesulfonate (ZI) was synthesized by literature procedure [20].

#### 2.1.2 Polymerization and hydrogenation of 5-phenyl-2-norbornene

Hydrated poly(phenyl norbornene) HPPNB was synthesized by one-pot sequential ring-opening metathesis polymerization and hydrogenation similar to literature procedure [21] as follows (Scheme 1). The first-generation Grubbs catalyst (24·mg, 2.9·10^−5^ mol) of in 3 ml of *p*-xylene was added to a solution of 5-phenyl-2-norbornene (15.0 g, 88 mmol) in 300 ml of dry *p*-xylene to initiate polymerization. The reaction mixture was stirred for 2 h. Each time the reaction mixture became so viscous that stirring was difficult, it was diluted with 50 ml of dry xylene (total volume: 200 ml). The reaction was terminated by addition of vinyl ethyl ether. Then p-toluenesulfonyl hydrazide (66.0 g 0.35 mol) was added and the mixture was stirring under reflux for 4 hours. The resulting solution was decanted to remove unreacted p-toluenesulfonyl hydrazide and precipitated into methanol. The product was filtered, washed three times with methanol and dried under vacuum. The polymer was re-precipitated twice from toluene to methanol and dried under vacuum at 40 °C to constant weight. Yield: 13.2 g (87%).

#### 2.1.3 Sulfonation reaction

Sulfonation reaction was carried out by propionic sulfate under homogeneous condition similar to that described for polystyrene-based block copolymers [22] in dichloromethane.

Propionic anhydride (7.25 g, 56 mmol) was added to 15 ml of dichloromethane cooled to 0 °C. To the solution 4.55 g of 98% sulfuric acid was added dropwise at such a rate that the temperature of the solution did not exceed 5 °C. The sulfonating mixture was added to a solution of 4.00 g of HPPNB in 60 ml of dichloromethane preheated to 40 °C on oil bath. The mixture was stirred under argon at 40 °C for 3h. The resulting gelatinous mixture was poured into 100 ml of 2-propanol to terminate sulfonation. All volatiles were isolated on rotary evaporator under vacuum at 60 °C. The residue was transferred to a flask contains dimethyl sulfoxide (50 ml). The mixture was stirred at 150 °C for 4 h. The resulting solution was cooled to room temperature and poured into 400 ml of diethyl ether. The precipitated SHPPhNB was filtered and washed 3 times with diethyl ether and dried under vacuum at 60 °C for 12 h.

#### 2.1.4 Synthesis of polymer with imidazolium and 1-methylimidazolium cation

To the mixture of SHPPhNB (2.0 g) and imidazole (0.54 g, 7.9 mmol) or 1-methylimidazole (0.65 g, 7.9 mmol) was dimethylformamide (50 ml) was added. The reaction mixture was stirred at 140 °C until it became homogeneous and then it was cooled to room temperature. The clear brown solution was poured into 300 ml of diethyl ether. After filtration and washing with 2 × 50 ml of diethyl ether SHPPhNB-Im or SHPPhNB-MIm was dissolved in 30 ml of methanol. The solution was poured again into diethyl ether. And the procedure was repeated once more time. Precipitated polymer was dried under vacuum.

### 2.2 Polymers characterization

NMR spectra were recorded in deuterated chloroform for non-ionic compounds (Fig. 1, a) and in deuterated dimethyl sulfoxide for sulfonated polymers (Fig. 1c–d) at 303K.

### 2.3 Membrane preparation and characterization

#### 2.3.1 Polyelectrolyte membrane preparation

The polymer membranes were prepared by solution casting method as follows. A total amount of 1.0 g of sulfonated polymer or its mixture with IL or IL and ZI was dissolved in dimethyl sulfoxide at 140 °C on a hot plate at 1400 rpm for 6 h. The mixtures were degassed under vacuum prior to casting. Resulting homogeneous solutions were cast on a Petri dishes (Ø 95mm) and left to dry in oven at 80 °C for 12 h. Then the films were stripped from the dishes and weighted to control full removal of the solvent. Five to seven pieces (∼20 × 20 mm) were cut from each membrane for testing. The ionic conductivity of the polymer membranes was determined by the ac complex impedance technique over the frequency range from 0.1 Hz to 5 MHz using a Р-45Х potentiostat/galvanostat equipped with FRA-24 M module (Electrochemical Instruments). The samples were sandwiched between symmetrical cells containing two coin-shaped steel electrodes with area of A (0.281 cm^2^) at the constant potential of 5 mV to measure membrane impedance, Z (Ω). The thickness of each sample L (cm) was measured prior to test with a micrometer. The conductivity (σ, S/cm) was then calculated from the equation: σ = L/(Z × A). The average ionic conductivities and standard deviation were calculated using Microsoft Excel (Table 1).

#### 2.3.2 PVDF blend membrane preparation

The membranes as a blend with PVDF were prepared by casting of the solutions prepared by dissolving 750 mg of PVDF, 375 mg of ionic polymer and 375 mg of IL in DMF at 120 °C. The mixtures were degassed under vacuum prior to casting. Resulting homogeneous solutions were cast on a Petri dishes (Ø 105 mm) and left to dry in oven at 80 °C for 12 h. Then the films were stripped from the dishes and weighted to control full removal of the solvent.

#### 2.3.3 Scanning electron microscopy of the membranes

Membranes were cut into square pieces (~ 1 × 1 cm). Both surfaces had been cleaned using a compressed gas duster before gold was sputter-coated on them. To observe the morphology of the cross section, membranes were fractured in liquid nitrogen. SEM images were obtained using backscattered electron (BSE) and secondary electron (SE) detectors (Fig. 4).
